# Masked or Disguised Intermittents

**Published:** 1849-09

**Authors:** T. Bullard


THE NORTH-WESTERN

MEDICAL AND SURGICAL JOURNAL

VOL. II.] SEPTEMBER, 1849. [No. 3.

### Part I.—Original Communications.

### ARTICLE I.

*Masked or Disguised Intermittents.* A paper read before the Indianapolis Medical Society, by T. BULLARD, M. D.

MR. PRESIDENT AND GENTLEMEN:—

I have chosen this topic for this evenings disscussion, not because it is in itself new, or that I believed I could present any thing original or novel worthy of your consideration, but because I have thought it a neglected, although to us and our patients oftentimes a vastly important subject, and with the hope, I may add, of eliciting such views and experience as might not be devoid of interest.

Among the causes of disease in the Mississippi Valley stands most conspicious that which gives origin to our more common forms of fever, and, indeed, the type of by far the greatest amount of disease. The nature of this cause I do not now propose to discuss. This, as yet, intangible, invisible, agent has been variously denominated marsh miasm, miasmata or malaria. Chemistry has not, as yet, been able to grasp it; the eye aided by the most powerful microscope has not detected it. We admit the existence of a peculiar morbific cause residing chiefly in alluvial or marshy districts, because we see effects manifested there, not otherwise accounted for. These effects are witnessed alike on the banks and through the valleys of our rivers, as well as on the plains and Savannahs of the South: in the bogs and fens around the City of the Czar, or in the Pontine marshes under the bright skies of Italy. But in order to appreciate its influence in those anomalous cases which I intend to present, let us glance at those more common forms of disease, in the production of which its power has been either acknowledged by the profession at large, or claimed and sustained by weight of authority not easily to be refuted.

Most of our authors admit that malaria is the remote or primary exciting cause of intermittent fever, and of periodical diseases in general. I am aware that Bell and others have objected to this theory, but facts and the weight of testimony seem still to be in its favor. But does malaria stop here as an essential cause of disease? What are our remittent fevers but imperfect instances of the great intermittent type modified either by a more concentrated poison or complication induced by its action.

Dr. Condie says:—

"Billious remittent fever is closely allied in its nature, the localities in which it chiefly prevails, and in many of its phenomena, to intermittent fever, of which by many it is considered a mere modification." Dr. Brown in Cycl. of Practical Medicine also, "presumes that a more intense operation of the same morbific cause required for the production of intermittent fever, engenders remittent, and the more violent the latter, the less perceptible the remission."

It is possible also, I think, that other fevers prevailing in miasmatic regions, and variously denominated by the books as typhus, typhoid, and continued, may become so modified by the action of malaria, that for practical purposes, at least, they merge into that numerous family of intermittent, whose parentage is due to this same poison, Perhaps, Gentlemen, I shall be considered as "breaking ground," but it is not so, other ploughmen have been before me; Dr. Mitchell, speaking of typhus and congestive fevers, says: —

"The position I assume here is plainly and boldly this; there is but one feature or element in either of the fevers named, that is essential to its pathology, and that feature, property, or element, bows before the sway of sulphate of quinine, and for this reason only we cure the patient."

He then gives his success in typhoid fever with large doses of quinine in cases where "do nothing" would have been the motto inscribed on the bed of the patients by those who say the disease will run its course, and that is sometimes six weeks, and all we can do is to see you safely on, yes "on" oftentimes to the grave. Nor is the Dr. alone in this view of the subject, for Dr. McCulloch and others maintain the same views. But without entering the disputed territory there is enough conceded dominion in which malaria reigns in unquestioned power, for my present purpose. With such a cause in action, then, we should not be surprised at great diversity in results and effects, when we consider the character of the subject upon which it acts, difference in age, sex, constitution, temperament, and locality of habitation &c., for except with the condition "*cœteris paribus.*" added, like causes rarely produce exactly in all points like results. And thus in intermittents, in practice we find a great variety of anomalous, masked or disguised forms of disease, yet having origin in a common cause.

Among our more systematic authors this subject is not mentioned at all by some, and barely alluded to by others; but our practical periodicals teem with varied and interesting cases. Dr. Brown in his article on intermittent fever in Dunglinson's Cyclopædia as he says himself, gives little more than a list of the various forms of masked intermittents. He divides them into inflammatory and nervous. Of the first, a part of his list consists of examples of pneumonia, pleuritis, carditis, otitis, peritonitis, ophthalmia, urticaria, erysipelas, rheumatism, gout, epistaxis, odontalgia, cephalalgia, meningitis diarrhœa, &c. Of the nervous, the most remarkable are asthma, periodical hysteria and epilepsy, intermitting deafness, convulsions, blindness, dumbness &c. Dr Brown quotes authorities, and names type, whether quotidian or not. Among others he quotes from an author writing in 1672, a quaint description of periodical sneezing, three paroxysms every evening and each paroxysm comprising 300 sneezes, also, a case of tertian eructation at the rate of 300 eructations per hour, examples of palsy and hemiplegia &c. But neuralgia is by far, he remarks, the most common form of masked intermittents. The first examples of this "mis-direction of the paroxysm," which drew my attention more particularly to this subject, were cases of diarrhœa: some of you may remember there were cases of this character in the Spring and Summer of 1845, in this vicinity. Then I was surprised to find that although the secretions had apparently been restored, and patients convalescent one day, yet on the second or third, or, perhaps, on the evening of the same day, there would be a recurrence of the diarrhœa as marked in its return as that of an ague, and yet no chill perhaps, and little or no fever. Believing this periodicity must depend upon the action of the same cause, which in others at the same time induced ague, I gave quinine as I would in a regular intermittent, and fortunately for our patients, if not for the Ledger, such cases were discharged in much less time than under ordinary treatment for idiopathic cases of the disease. I doubt not others present can bear witness to many similar instances. The same has been true of some cases of Dysentery the past season. But a few weeks since, I attended a marked case of this character. Dysentery supervened upon an ordinary attack of Measles; the patient, a girl of 6 or 7 years of age had frequent and bloody stools with great tormina and some fever: I treated the case for some days as I would an ordinary instance of idiopathic Dysentery: every morning there seemed a decided improvement; one or two dark consistent evacuations, with little or no griping, very little blood and no fever. I believed my patient at such times convalescing; notwithstanding, in the evening the disease recurred with increased violence. In short, I became exceedingly anxious for the little patient. I had given Quinine in small doses for two or three mornings, to sustain my patient, but this did not avail to arrest the recurrence. I now gave the sulphate in large doses in the morning, and she had a much better evening and night. I repeated the Quinine the following morning; the tormina and bloody stools did not return and my patient rapidly convalesced. I am sure those 35 or 40 grains of Quinine saved my patient, and that they did so, not by restoring the secretions—my former treatment had apparently accomplished this—but by arresting at once the masked intermittent which to all appearances was fast hurrying her to the grave. Nor is such a case as this new; on the contrary, Morton describes an epidemic Dysentery prevailing in London in the middle of the 17th Century, in which he says: "The affection was only a symptom of the remittent fever, with distinct remissions and exacerbations every first or second day." To this might be added the testimony of Sydenham, Willis, Mosley, Roederer, Clark and many others—Says Mnyhorn, "when the fever and gripes were regularly exasperated every day or every other day, at stated periods, the Bark has often effectually put a stop to both." Dr. Wilson in his work on China, says: "In truth the flux ought to be considered a constituent part of the periodic fever, rather than an independent malady in most instances.'

Another form to which I will allude is Intermittent Hemiplegia, Cullen, mentions this as 'paralysis intermittens.' An interesting case of this character is recorded by Dr Elliotson, and inserted in the "Western Journal" 16 years ago. The man was 48 years of age, paroxysms came on about 10 o'clock every third or fourth day, lasting 3 or 4 hours. He had been subject to the affection at intervals for two years and a half: he had been in the East and West Indies. The Dr. says: "I do not doubt that this was the effect of malaria, that his hemiplegia was a form of ague. I will not quarrel about words, you might say it was not ague because unattended with shivering, fever, sweating, &c., but I have no doubt it was as much the effect of malaria as ague is." Acting on this belief the Dr. gave Quinine in large doses, considering the but recent introduction of the article into use, 40 grs. in 24 hours, 10 grn. doses. On the patient having a relapse 3 months after, the Dr. gave him 60 grs. in 24 hours, 15 grn doses, the Paralysis ceased and the patient improved in general health. Who can doubt, had this man been treated for an organic disease, for effusion or compression, foı instance, but that there would have been an entire failure to say the least of it! Was it not as the Author remarked, "an affair of function induced by a particular poison?"

*M. Mendiere*, in the *Revue Medicale*, has published several cases of obstinate Hiccup, associated with, or dependent upon affections of the digestive organs, originating in miasmatic influence, which he cured promptly and decisively by the free use of Quinine, after they had resisted every other mode of treatment. Dr. Hogan, of the U. S. Army, says he has found the paroxysm of Asthma, is as tractable to the influence of the Sulphate as the slightest quotidian." Dr. Simon, in a French Journal, related three cases of Asthma rapidly cured by the same medication. Many instances might be quoted of Neuralgic and Hysteric affections assuming a periodic character, which when observed and treated by antiperiodic remedies, were found to yield as readily as common ague.

But it is not necessary for my purpose. Dr. McCulloch says he has seen at least four cases of absolute and pure *Mania* of most distinct tertian and quotidian forms which had become the subject of gross mistreatment. "The exacerbations were in all as distinct as those of a common intermittent would have been, and as regular, while the two tertian cases left a day intermediate of the most entire sanity of mind, the period of the paroxysm being a perfect mania and not a febrile delirium, and while in fact no marks of fevers could be discovered, and in one of these at least, the cause was as easily, as it was distinctly traced. In one the disorder had commenced, as a common tertian, which *seemed* to have entirely disappeared to be exchanged for the mania, while of the other, whatever my suspicions were, I could not procure a distinct previous history, from the inattention and ignorance of the friends and patient. In the quotidians not only were there accessory, if obscure, symptoms of intermittent to be found on an accurate inquiry in both of them, but while the original cause in one of them was distinctly traced, so was the truth of the conclusion confirmed by the success of the method of cure which was adopted, after great maltreatment." There are certain affections of the eye which are modified, at least, by malaria, and which if not recognized a "depletory treatment may be persevered in for a most unnecessary length of time, to the great prejudice of the patients general health and the decided aggravation of the local malady." I had such a case of ophthalmia not long since, general treatment and local appliances for a time seemed to encourage the hope of a speedy cure, but it held on. Some days the patient would say his eyes felt about as well as ever; in a day or two, back again would he come, like a bad penny—I began to open my own eyes, and enquired if his eyes were not worse every other day. Did not know, had not observed. I satisfied myself it was so, and gave Quinine in large doses, and the result exceeded my most sanguine expectations. Perhaps it might be a question of ethics whether I ought to have charged him anything for my former treatment. Hoffman, Morton, Van Swieten, &c., notice many interesting cases of Ophthalmia, which, after resisting all antiphlogistic treatment, speedily gave way to the free use of antiperiodics. As it has been remarked, the difficulty lies not in treatment, but in the diagnosis of the disease, and I believe much of this will disappear when we shall become fully impressed with an adequate idea of the influence which malaria exerts, in giving character to our diseases. Not that I desire in any sense to be regarded as a routinist, or that I would willingly select any class of articles from our "Materia Medica," and make them a *hobby*.

I had sketched the history of a very curious case which came under the observation of my partner and myself, the past season, but lest I should become tedious, I will only allude to it. At the time, I had never seen a similar case, either in practice, or a description in any author, and I confess I was at a loss how to christen it! Since, I have observed two, somewhat similar, described under the term of "Flat Vision." Our patient could only see the half of an object in its natural proportions, and that at but a short distance and dimly; the other, appeared wanting or perfectly flat.

This symptom was promptly removed by Quinine and other tonics, and the power of vision increased by them and the influence of Galvanism, conjoined. Without alluding to intermittent palpitations, complications attending our pneumonias, &c., &c., I have thus simply selected specimens from a great variety.

In taking such a view of the *possible* effects of malaria, I would not be understood as claiming that all, or perhaps any, of these various forms of disease are *always* necessarily, either dependent upon miasmatic causes, or cured alone by antiperiodic remedies. On the contrary, it is because there are forms of these multitudinous affections, often associated with, or dependent upon organic lesion, inflammation or functional derangement, for which these "masked" varieties are liable to be mistaken, that we have thus endeavored to show the influence of so subtle an agent, and the guise it may assume. Nor in regard to the treatment, which, by the way, has been only touched upon incidentally, do we claim that antiperiodics should be the sole means of cure, in these cases, more than in common ague: and probably local inflammation &c., will often call for more energetic auxiliary treatment in these forms than in that of their regular type. Quinine, for instance, will alone often arrest the intermittent, whether open or disguised, but other and auxiliary medication may be required to restore to healthy action the various functions of the organism. And as the type of our fevers varies from time to time, now demanding the free use of the Lancet, &c, again rarely if at all, so do individual cases of any given disease require more or less modification in treatment; yet admitting the necessity of auxiliary and preparatory treatment, the remedy which seems almost an antidote to a poison of such power, the fatal results of which we yearly behold, should be given at the earliest practicable moment. I know there is authority, even that of an Eberle, for waiting for a number of paroxysms to pass before the anti-periodic is given; but I ask in the name of humanity, if he had seen as we have, fatal congestions supervene in the 2d or 3d paroxysm, of what seemed to patient, friends, and to the attending physician but simple ague, would he, or any other honest man, dare advise delay till the possible advent of the 6th or 7th attack? But supposing there was no such contingency, is the amount of suffering in each attack and the acknowledged detriment to the patient's system, of no account? or is it to be balanced by the Physicians augmented bill? In regard to the choice of the antiperiodic in these masked intermittents there are cases especially of Neuralgic character, where arsenic and some other agents will succeed, when Quinine or Bark fails. But I apprehend that where the sulphate has disappointed, most generally, some local complication has been overlooked, or the article has been *dosed* out in by far too minute doses. It has not been half tried until large, and if necessary, *heroic* doses have been given. It should never be said to have failed until the constitutional effects, the Quinine intoxication, has been induced, and although it may do so even after this has been secured owing to an imperfect remission, or the state of some one or more organs at the time, yet I believe this should not be regarded as proof of its failure, and that in a subsequent stage of the disease, a more perfect intermission or remission may be obtained, when the disease, as Mitchel expresses it, "will bow to the potent sway of the Sulphate of Quinine."

